# Evolutionary patterns of diadromy in fishes: more than a transitional state between marine and freshwater

**DOI:** 10.1186/s12862-019-1492-2

**Published:** 2019-08-14

**Authors:** Joel B. Corush

**Affiliations:** Department of Ecology and Evolutionary Biology, University of Tennessee, 569 Dabney Hall, Knoxville, TN 37996 USA

**Keywords:** Anadromy, Catadromy, Amphidromy, Habitat transition, Multiple state speciation and extinction (MuSSE), Alternative life history

## Abstract

**Background:**

Across the tree of life there are numerous evolutionary transitions between different habitats (i.e., aquatic and terrestrial or marine and freshwater). Many of these dramatic evolutionary shifts parallel developmental shifts that require physiological, anatomical and behavioral changes for survival and reproduction. Diadromy (scheduled movement between marine and freshwater) has been characterized as a behavior that acts as an evolutionary intermediate state between marine and freshwater environments, implying that diadromous lineages are evolutionarily transient. This hypothesis comes with assumptions regarding the rates of evolutionary transitions in and out of diadromy as well as rates of speciation and extinction in diadromous fishes.

**Results:**

Based on a published phylogeny of 7822 species of ray-finned fishes, state speciation and extinction models of evolutionary transition between marine, freshwater, and diadromous species suggest transition rates out of diadromy are 5–100 times higher that transition between marine and freshwater or into diadromy. Additionally, high speciation and low extinction rates separate diadromous fishes from marine and freshwater species. As a result, net diversification (net diversification = speciation – extinction) is about 7–40 times higher in diadromous fishes compared to freshwater and marine respectively. Together the transition, speciation, and extinction rates suggest diadromy is the least stable of the three states.

**Conclusion:**

Evolutionary transitions to diadromy are rare in fishes. However, once established, diversification rates in diadromous lineages are high compared to both marine and freshwater species. Diadromous lineages tend to be more transient than marine or freshwater lineages and are found to give rise to marine and freshwater specialists in addition to diadromous descendants. Although diadromy is not a necessary evolutionary intermediate between marine and freshwater, these results support the interpretation of diadromy as an important, occasionally intermediate state, that contributes to biodiversity in fishes in all environments. This evolutionary instability of diadromous lineages is counteracted by their relatively high diversification rates. These findings highlight the importance of integrating the dynamics of diversification and major evolutionary transitions for understanding macroevolutionary patterns.

**Electronic supplementary material:**

The online version of this article (10.1186/s12862-019-1492-2) contains supplementary material, which is available to authorized users.

## Background

Major evolutionary transitions between different habitat types are seen across the tree of life. Transitions between water and land [1], flightlessness and flight [2], and marine and freshwater [3] all require major behavioral, physiological and morphological changes, yet each transition has occurred numerous times [1–9]. Explaining the high frequency of these transitions is an important challenge for evolutionary biologists, as these transitions allow species to move into niches not previously occupied. Identifying specific individuals or species that make these transitions as part of their life history could be key in understanding the evolutionary transitions. Life histories in which these major transitions occur ontogenetically are found in many taxonomic groups, including salamanders that reproduce in water and live on land; fishes, crabs and snails that migrate between marine and freshwater; sea turtles that are born on land and hatchlings immediately move to the ocean; and many semi-aquatic insects, such as dragonflies, that have an aquatic larval stage but a terrestrial adulthood with flight. While it is convenient to find examples of species with transitional life histories at the evolutionary transition between lineages occupying different habitats, explicit tests are needed to understand if transitional life histories are acting as an evolutionary intermediate (i.e., a life history that occurs as an intermediate between two other life histories such as semiterrestrial in the case of aquatic ➔ semiterrestrial ➔ terrestrial evolutionary transitions). Here I test the idea that the fishes that migrate between marine and freshwater habitats within their lifetime are also the evolutionary intermediates between completely marine and completely freshwater lineages.

**Fig. 1:**
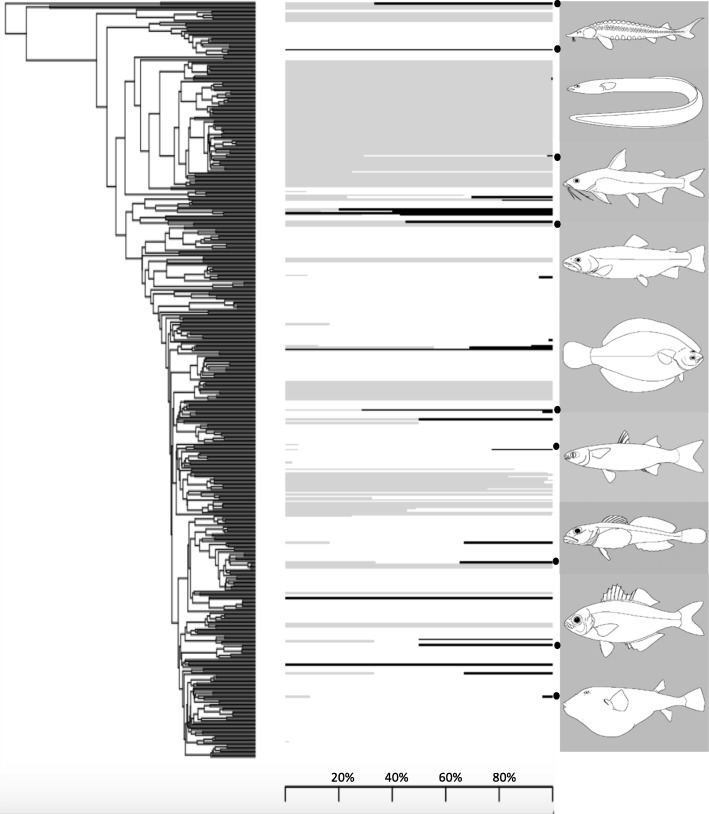
Relative distribution of diadromy in fishes. Phylogeny from Rabosky et al. [12] pruned to the family level. Horizontal lines represent the percent of freshwater (gray), marine (white), and diadromous (black) species from each family represented in the original tree. Note that percentages are based only on species used in the original phylogeny. For example, in the family Pseudaphritidae two species are recognized, one diadromous and one marine. This study only incorporates *Pseudaphritis urvillii*, the diadromous species, so the horizontal line for the family Pseudaphritidae is completely black despite the only 50% of the family being diadromous. Each family is represented by only one tip and placement of non-monophyletic families was chosen based on the placement of the majority of its species. Dots next to pictures represent placement of select families as a general reference. Families represented are, from top to bottom, Acipenseridae, Anguillidae, Ariidae, Salmonidae, Pleuronectidae, Mugilidae, Cottidae, Kuhliidae, Tetraodontidae. Pictures from Fishbase [54]

Among fishes, one of the most dramatic changes in habitat type is between marine and freshwater. Diadromy, or the migration between marine and freshwater during a particular life stage [4, 6], requires numerous physiological changes in osmotic and ionic regulation and differs from euryhaline fishes that move across a wide gradient of salinity with regularity [4, 6, 10, 11]. This scheduled movement between environments is of ecological and evolutionary interest, as it is rare (0.8% of the 32,000 species of ray-finned fishes), yet phylogenetically widespread (8% of the nearly 500 families of fishes) [6] (Fig. 1). In addition, diadromy is observed in other animals including snails [7], prawns [13], and crabs [14, 15].

Multiple hypotheses have been proposed to explain the evolutionary significance of diadromy [5, 8, 9, 16]. Many studies focus on the evolutionary transitions in and out of diadromy and propose that diadromy acts as an intermediate stage between marine life histories and freshwater life histories. While the proposed mechanisms range from resource availability for adults based on latitudinal variation in net primary productivity of environments [5, 8] to moving into a safe site for spawning [9], each of these hypotheses suggests a similar evolutionary trajectory: A marine species has individuals or lineages that venture into freshwater (be it during adulthood or spawning) and transition into a diadromous species. Over time, lineages of the diadromous species stop returning to marine systems, becoming completely freshwater due to some advantage(s) of the new environment (i.e., marine ➔ diadromy ➔ freshwater). Similar, arguments have been constructed in the opposite direction (i.e., freshwater ➔ diadromy ➔ marine) [5, 8]. The natural world provides many examples of evolutionary transitions where diadromy acts as an intermediate, such as sticklebacks (Gasterosteidae), lampreys (Petromyzontiformes), salmonids (Salmonidae), and shads (Clupeidae) [6, 17, 18]. While these examples support the directionality in the previously proposed hypotheses [3, 18], there are also examples of evolutionary transitions involving diadromous species not acting as an intermediate such as freshwater eels (Anguillidae), herrings (Clupeidae), and flagtails (Kuhliidae) [3, 6, 17, 19, 20]. Some of these examples, such as the flagtails, transition from marine to diadromy and back to marine [20]. There are also a number of putative examples of transitions directly between marine and freshwater [21–24]. These numerous exceptions suggest that diadromy might be more than an evolutionary intermediate.

Modern phylogenetic comparative methods can utilize time calibrated phylogenies to estimate rates of evolutionary transition between life histories (*q*), speciation (λ) and extinction (μ). These rates can then be incorporated into the hypotheses of diadromy as an evolutionary intermediate. In addition to testing the rates of evolutionary transitions in and out of diadromy, if diadromy is solely a transitional form, it would be expected that diadromous species would show little variation in speciation and extinction rates compared to marine and freshwater species. Drastic differences in speciation and/or extinction rates would suggest that the life history of diadromy affects the net diversification rate (*r =* λ - μ) of fishes. In fact, diadromous behavior has been proposed to have seemingly conflicting effects on genetic structure and the two components of net diversification [6, 25]. On one hand, diadromous migration between different river catchments is expected to decrease population structure compared to completely freshwater species which cannot move between river systems via the ocean. This should decrease the risk of extinction due to unfavorable habitat changes or stochastic events that would wipe out a completely freshwater species that is fully restricted to that freshwater system. At the same time diadromy could decrease the rate of speciation due to increased gene flow between isolated river systems via migration into and back out of the ocean. On the other hand, diadromous migrations are expected to increase the geographic range of a species compared to completely freshwater taxa, potentially increasing isolation-by-distance and changing the selection pressure put on a population as it expands into new habitats. This might increase rates of ecological speciation via local adaptation or vicariant speciation via landlocking or other barriers to dispersal which can disrupt widely distributed species [6, 13, 25, 26]. There are also conflicting patterns compared to marine species. Diadromous species might have increased population subdivision due to even a small restriction to freshwater habitats for a portion of their lives. This should increase speciation rates in diadromous species relative to marine species (although some marine reef associated fishes have extremely limited dispersal and a high level of population structure [27–29]). Comparative analyses on a few select taxa suggest that diadromous fishes have an intermediate level of population structure compared to the more structured freshwater and less structured marine species [30]. Based on the compounding factors including range size shifts, population differentiation, and geneflow in each instance of diadromy, diversification patterns should vary from marine and freshwater suggesting that diadromy may be responsible for shifts in diversification rates, not just a transitional phase. Coupling the assumptions about habitat use associated with transition patterns and the assumptions about speciation and extinction associated with population dynamics may shed light on the true nature of diadromy.

Previous phylogenetic analyses have addressed the evolutionary transitions in and out of diadromy but tend to focus on specific families or orders of fishes [3, 20, 23, 31–34]. However, these studies can suffer from low statistical power owing to small numbers of evolutionary transitions and little time for speciation and extinction to occur (e.g., Feutry et al. [20] examine one transition into diadromy and one transition out of diadromy). Some clade-specific studies find no significant difference between marine and freshwater transition rates [24], while other fish groups show considerably more transitions from marine to freshwater compared to the reverse [22]. Some groups have few transitions into diadromy [6, 20, 34], while others have numerous transitions both in and out of diadromy [3, 6]. These studies also tend to focus on clades with relatively high prevalence of diadromy. Thus, they might provide insight into the timing and mechanisms of particular transitions by examining a few species within an ecological context but have limited scope for generalization to all fishes. To better assess the evolutionary significance of diadromy, this study uses the most complete and time-calibrated tree of 7822 fishes constructed using 13 genes and 60 fossils for time calibration [12, 35] coupled with MuSSE (Multiple State Speciation and Extinction) [36], a well-established method, to better elucidate the diversification within and transition between three states (i.e., marine, freshwater and diadromous) across ray-finned fishes. The MuSSE model uses a time-calibrated phylogeny to generate rate estimations of speciation (λ) and extinction (μ) within each of the three states, as well as rates of transition between each state (*q*).

**Fig. 2:**
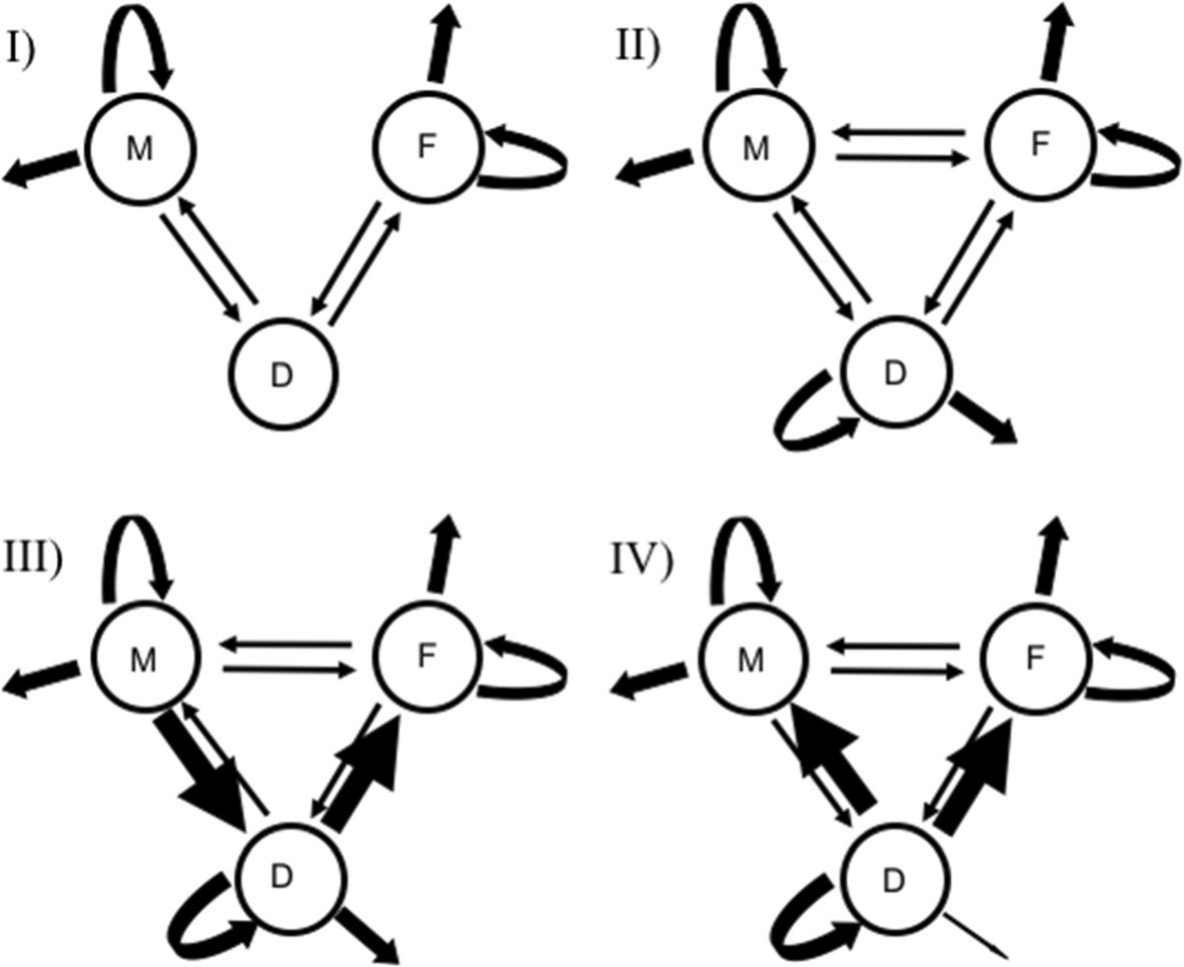
Illustrated hypotheses of the evolution of diadromy. Circles represent marine (M), freshwater (F) and diadromous stages (D). Arrows between states represent transitions (*q*_ij_), arrows leaving and returning to the same state represent speciation events (λ_i_), and arrows leading away from each state represent extinction (μ_i_). Size of arrows within each category (speciation, extinction and transitions) are representative of the rate variation, but not sized to scale. I) Model hypothesizing that diadromy is a transition state between marine and freshwater. This hypothesis should be better supported if diadromy prevails only as an evolutionary stepping stone to other character states. II) Hypothetical model where diadromy is evolutionarily stable and its prevalence is influenced by variation in rates of speciation and extinction. III) Hypothesis showing movement between marine and freshwater utilizing diadromy as a stepping stone, while still allowing for direct movement between marine and freshwater. IV) Optimal models based on MuSSE results

The first aim of this study is to test the hypothesis that diadromy is an intermediate evolutionary stage between marine and freshwater lifestyles (Fig. 2), including a test of the extreme scenario that all transitions between marine and freshwater have to go through diadromy (Fig. 2I). This extreme scenario is contradicted by cases of apparent transition between marine and freshwater without evidence of a diadromous intermediate [22, 24]; however, large scale phylogenetic analysis could support the existence of diadromy as a temporary or “hidden” transitional state. A more realistic null model is one with all transition rates being similar regardless of the direction (Fig. 2II). Additionally, if marine ➔ diadromy ➔ freshwater happens at a different rate from freshwater ➔ diadromy ➔ marine, shifts in rates based on directionality should be observed (Fig. 2III).

The second aim of this study is to test the hypothesis that, even though not all transitions between marine and freshwater go through diadromy, and regardless of the directionality of transition in and out of diadromy, diadromy only acts as a transient stage, resulting in a net diversification rate in diadromous fishes that is lower than either marine or freshwater fishes. This hypothesis suggests that partial movement into new habitats should not result in speciation or extinction, but instead transition out of diadromy (Fig. 2I). An alternative hypothesis predicts an intermediate or higher diversification rate arising from the expected effects of diadromy on population structure, range size, gene flow and extinction risk. The two aims of this study should also be looked at together. The idea that diadromy is primarily a transitional state would be supported if transitions involving diadromy are quantitatively dominant and diversification of diadromous lineages are relatively unimportant. It is important to note that both hypotheses are strictly pattern-oriented assessments of rates and do not address causality in the system. As might be the case in family or order specific studies, no attempt is made here to test why or how diadromy evolves. This study focuses on the quantitative importance of diadromy by assessing transition, speciation, and extinction rates of marine, diadromous and freshwater fishes.

## Results

### Phylogenetic hypothesis testing

**Fig. 3:**
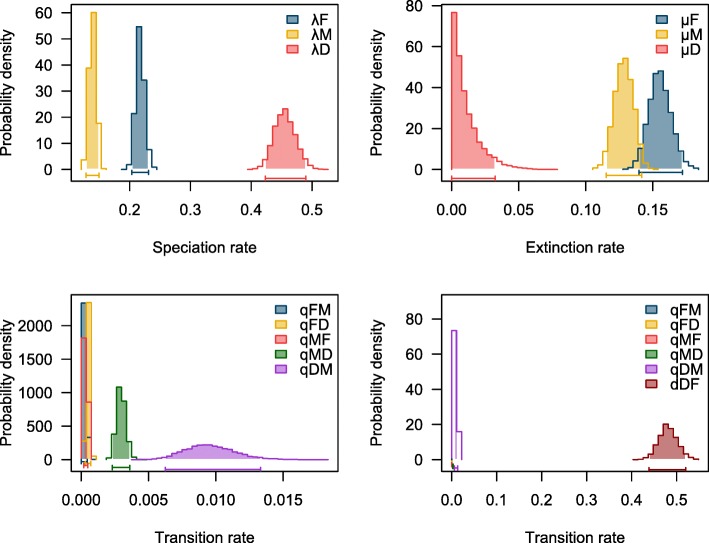
Posterior probability distributions for the speciation (λ_F_, λ_M_, and λ_D_), extinction (μ_F_, μ_M_, and μ_D_), and transition (*q*_FM_, *q*_FD_, *q*_MD_, *q*_MF_, *q*_DF_, and q_DM_) rates inferred by the MuSSE. Distributions are based on 10,000 generations with a burn-in of the 10%

The transition rate from diadromy to freshwater (*q*_DF_ = 9.37 × 10^− 3^) is higher than transitions in the reverse direction (*q*_FD_ = 4.98 × 10^− 4^) and the transition rate from diadromy to marine (*q*_DM_ = 4.81 × 10^− 1^) are also higher than transitions in the reverse direction (*q*_MD_ = 2.87 × 10^− 3^). The highest transition rate overall reflected movement from diadromous to marine lifestyles (Table 1A). Transition rates inferred from fitDiscrete analyses corroborated the MuSSE results showing high transition rates out of diadromy. The ARD model was best fit for both the two state and three state models (Table 1B). Both of the best fit models inferred transition rates out of diadromy that were larger than any other transition rate. Regardless of whether the “unknown” species were assigned to marine or freshwater states, the estimated parameter rates from MuSSE remained similar (Additional file 3). Posterior distributions from MuSSE show strong variation between diadromous and both non-diadromous states for both speciation and extinction rates as well as transition out of diadromy as opposed to all other transitions (Fig. 3). BiSSE and HiSSE justified the assumption of “diadromy” as a group for MuSSE analysis. BiSSE and HiSSE analyses resulted in no support for a hidden state implying no sub groupings within diadromous fishes (i.e., each diadromous fish was grouped into the “diadromy-observed” state with > 98% likelihood) (Additional file 4). HiSSE did assign non-diadromous fishes into two rate categories that did not match up with character assignment of marine and freshwater species. Because of the differences in the nature of the three state MuSSE (i.e., diadromous, marine and freshwater) and the two-state BiSSE and HiSSE models (i.e., diadromous vs. non-diadromous), rate comparison cannot be made between the two model types. Additionally, neither BiSSE nor HiSSE can assess diadromy with respect to its role as a transitional state between marine and freshwater because they are combined into one state.

**Table 1:**
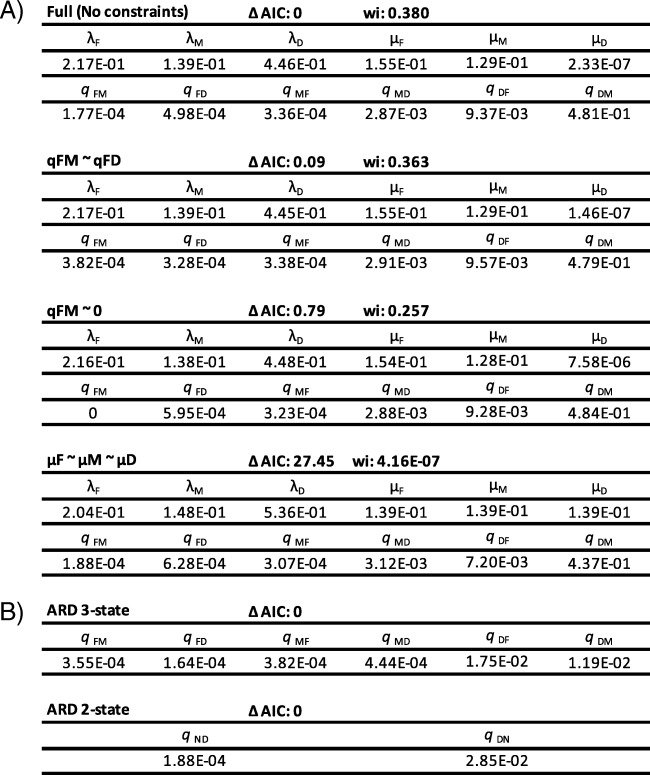
Output parameters for key model in MuSSE and fitDiscrete analyses

Output parameters for A) Three best fit MuSSE models and model with constraints on extinction rates. Speciation rate (λ_i_), extinction rate (μ_i_), and transition rates (*q*_ij_) where _i_ and _j_ refer to the original and new states respectively. B) Best fit fitDiscrete models for both two and three state models

Based on MuSSE, the net diversification rate (*r* = λ – μ) in diadromous fishes (*r* = 0.446) is about an order of magnitude higher than in both marine (*r* = 0.01) and freshwater (*r* = 0.062) fishes (Table 1, Additional file 2). The observed pattern of freshwater fishes having a higher speciation and extinction rate compared to marine fishes is in line with estimates in New World silversides [31]. State Stability (*S*) was lowest for diadromy (*S*_D_ = 0.51 = 1–2.33e^− 7^-9.37e^− 3^-4.81e^− 1^) compared to marine (*S*_M_ = 0.87 = 1–1.29e^− 1^-3.36e^− 4^-2.87e^− 3^) and freshwater (*S*_F_ = 0.84 = 1–1.55e^− 1^-1.77e^− 4^-4.98e^− 4^).

Results indicate that diadromy is a rare state (low transition rates into diadromy) that, if obtained, can lead to rapid diversification (high speciation and low extinction rates) and large numbers of descendants specialized in either marine or freshwater (high transition rates out of diadromy) (Fig. 2IV). If the intermediate state hypotheses [5, 8] were true, we would expect the models constraining transition through diadromy (i.e., *q*_FD_~*q*_DM_, *q*_FM_~*q*_DM_) or those limiting movement between freshwater and marine (i.e., *q*_FM_~ 0 and/or *q*_MF_~ 0) to all be better fit models (Additional file 2). In the best-supported model, transitions occur between all states. The top models show highest transition levels while exiting diadromy (Fig. 2IV) and do not show patterns expected from simple interpretations of diadromy as an intermediate state (Fig. 2I, III). Additionally, no model in which the speciation and/or extinction rates in diadromy were constrained to equal marine and/or freshwater rates were well supported. High diversification rate suggest that some population dynamics may be affected by the ecological complexities of diadromy and that diadromy may have some other evolutionary significance. The high diversification rate is greatly impacted by a particularly low extinction rate in diadromy.

### Model adequacy testing

**Fig. 4:**
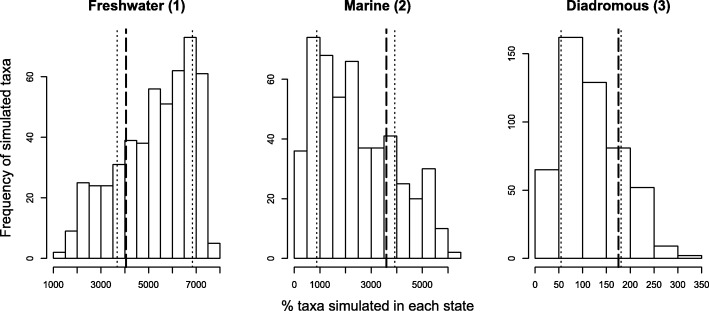
Simulated distribution of marine, freshwater, and diadromous fishes. Histograms of percent taxa in each state (freshwater, marine, and diadromous) from 500 simulated trees using the output parameters from the optimal MuSSE model. All trees were assigned marine as ancestral state. Thick dashed lines indicate the observed values for each state. Thin dotted lines indicate one standard deviation from the mean of simulated values

Simulations of 500 trees that used the output parameters of the MuSSE model resulted in a distribution of trait frequencies such that the observed values fell within one standard deviation of the mean of the simulated distribution (Fig. 4).

## Discussion

Substantial transition rates between marine and freshwater in the best fit model do not support the hypothesis that all transitions between marine and freshwater move through diadromy. The phylogeny includes many recent transitions, decreasing the likelihood that there is an unobserved diadromous ancestor required in the transition. The patterns observed are neither in support of equal rates across all transitions (Fig. 2II) nor diadromy as an intermediate with one-to-one transition rates from marine to diadromy to freshwater or the other way around (Fig. 2III). The highest transition rates in both the fitDiscrete and MuSSE analyses were those out of diadromy to both marine and freshwater, which is in part responsible for diadromy having the lowest stability (*S*_D_ = 0.51). Additionally, transitions from marine to diadromy were observed at an intermediate rate and those from freshwater to diadromy and between marine and freshwater showed the lowest rates. While transitions between marine and freshwater may move through diadromy, the evidence does not support labeling diadromy as a necessary transitional state. The hypothesis that even though not all transitions between marine and freshwater go through diadromy, and regardless of the directionality of transition in and out of diadromy, diadromy only acts as a transient stage is also not supported in light of high diversification rate observed in diadromy. This is a function of both high speciation and low extinction rates in diadromy. Instead, an evolutionary significance of diadromy may be that it acts as a potential source of diversity by allowing a lineage to enter a new habitat. This habitat transition may select for behavioral, anatomical or physiological variants that allow lineages to be successful in the new habitat or revert back to the ancestral habitat but with some variation from close relatives.

Many mechanisms could be responsible for the high rates of transition exiting diadromy including landlocked and oceanlocked populations. Freshwater populations can become isolated each time a set of random individuals becomes landlocked due to factors such as: a receding glacier forming isolated lakes, change in flow patterns of rivers, or the formation of natural dams due to tectonic processes. Oceanlocked species can result from a loss of suitable freshwater habitat in a species whose populations are geographically limited to ephemeral island streams. These high transition rates out of diadromy are illustrated by many examples of diadromous species with non-diadromous individuals, populations, or sister species [6, 18, 19, 37]. Species in which ocean- and landlocking has resulted in genetic isolation include alewives, galaxiids, gobies, sticklebacks, and lampreys [6, 18, 38]. With multiple ocean- or landlocking events and a wide enough distribution, multiple completely freshwater species, or completely marine species in the case of eels [19, 39], can emerge from a single diadromous species. Once species have diverged, fine-scale niche preferences can support a diversified clade in their newly occupied habitats [40]. The directionality of observed transitions suggests that diadromy may sometimes be an intermediate state between marine and freshwater life histories and sometimes a temporary state in which lineages persist before returning to their ancestral state. Many papers address the causes and mechanisms of these transitions [5, 8, 9, 20]; however, those are beyond the scope of this study.

The high net-diversification rate of diadromous fishes is somewhat unexpected due to the potential for dispersal (especially in species without a strong tendency to return to their natal sites), since these fishes are capable of movement between distant freshwater sources during their marine phase. A wide range of dispersal distances have been reported in diadromous fishes [26, 41, 42]. Comparisons in some groups, such as minnows, show that marine dispersal is associated with larger range [34] and in lampreys, dispersal decreases the isolation by distance over large areas [38]. The variation in dispersal time and distance is important in speciation and extinction rates. Short dispersal potential can result in a high level of isolation while still allowing for occasional propagules to venture out and form new populations with low levels of gene flow, a classic scenario for geographic speciation [43]. Long distance dispersal can result in less structured populations compared to poorly dispersing diadromous fishes but could increase the overall range of the species allowing for more opportunities for isolation and transitions out of diadromy, especially in species that exhibit facultative diadromy. These results contradict expectations that diadromy has an intermediate diversification rate associated with an intermediate level of gene flow and range size compared to marine and freshwater fishes. Instead, diadromous fishes have higher diversification rates than either freshwater or marine lineages. The estimated speciation rate is about twice that of freshwater species and four times that of marine species (Table 1). This might arise if diadromous species, rather than having intermediate population structure, experience the best of both worlds for speciation. That is, perhaps diadromous species tend to have large geographic ranges (more like marine species on average), but also high levels of subdivision and local adaptation (more like freshwater species on average). Even more striking, the estimated extinction rate is several orders of magnitude lower for diadromous vs non-diadromous lineages (Table 1). Taken together with the high transition rates, it appears that diadromous ancestors might often escape extinction by leaving relictual freshwater populations in the case of unfavorable conditions in the ocean, and likewise by persisting in the ocean when freshwater populations are extirpated.

### Observable patterns of diadromy

While the results presented here neither support nor refute the proposed hypothesis with respect to the causality of the evolution of diadromy, they do not conflict with the possibility of the transition patterns proposed by Gross et al. [5, 8] and Dodson et al. [9]. These results support a hypothesis that a high rate of transitions out of diadromy coupled with the high net diversification rate in diadromy leads to a single species becoming diadromous, diversifying and then many species returning to non-diadromous lives. To date, many existing phylogenetic studies have discussed particular clades that fit the pattern presented by the MuSSE analysis, but not the transitional state hypotheses. Using the flagtails as an example, Feutry et al. [20] show a marine ancestry that transitioned into diadromy, and once diadromy evolved, multiple speciation events occurred, giving rise to six extant diadromous species of *Kuhlia*. Then, presumably via the gradual transition of the partially diadromous *K. munda*, a transition back to a completely marine life history occurred. Of the diadromous species, half of them (i.e., *K. malo, K. rupestris* and *K. munda*) exhibit intermediate phenotypic variation and/or partially catadromous life histories. While extant species show only one well defined transition out of diadromy, the loss of total obligate diadromy in some of the diadromous species could be an additional transition out of diadromy. As pointed out by Feutry et al. [20], the idea proposed by Gross et al. [5, 8] that marine species in tropical regions utilize freshwater habitats because of the increased resource availability does not apply to small island streams that the *Kuhlia* typically inhabit, as they tend to be nutrient poor.

In salmonids, Alexandrou et al. [44] show a transition from freshwater to diadromy and back to freshwater within Coregoninae. This transition into diadromy has resulted in eight diadromous species. Nested within the diadromus *Coregonus* species are three freshwater species (*C. nigripinnis*, *C. zenithicus,* and *C. hoyi*). Nested in those three species is another diadromous species (*C. artedi*). Either *Coregonus artedi* was a second transition into diadromy after a transition back to freshwater, or transition from diadromy to freshwater occurred twice in *C. nigripinnis* and in the *C. zenithicus* and *C. hoyi* clade. However, inconsistencies in the placement of various taxa in *Coregonus* [45, 46] could drastically change the order of events with regards to transitions and any assumptions about the evolution of diadromy could be incorrect. If we are to assume the Crete-Lafreniere et al. [45] phylogeny is correct, additional transitions out of diadromy are observed: 1) The Ireland endemic *C. pollan* nested in the widespread *C. autumnalis,* and 2) A group of multiple species sharing a common ancestor with the polyphyletic *C. artedi* which inhabit the freshwater of the US Great Lakes species. Some of these transitions (e.g., freshwater to diadromy in temperate regions) do agree with Gross and some do not (e.g. diadromy to freshwater in temperate regions) [5, 8]. In addition to the Coregoninae clade, Alexandrou et al. [44] show two additional transitions out of diadromy within salmonids in the genera *Salmo* and *Salvelinus*. Both Alexandrou et al. and Crete-Lafreniere et al. produced phylogenies with many diadromous species arising after a transition from freshwater, and at least one, if not multiple transitions from diadromy to freshwater. These patterns coincide with the parameters of the best fit MuSSE model.

Within the Anguilliformes, one major transition from marine to diadromy has occurred in the freshwater eels of the genus *Anguilla* resulting in the 16 species and three subspecies [47]. In agreement with the prediction of Gross et al. [5, 8], we find marine ancestors transitioning to a diadromous life history in a tropical region. There is evidence that in tropical regions individuals of *Anguilla marmorata* [48], and in temperate regions *A. japonica* and *A. anguilla* [39], have reverted back to completely marine life histories. Although whether these individuals reproduce or are merely lost at sea remains unknown, the large scale marine ➔ diadromy ➔ marine scenario does not fit the hypotheses proposed by Gross et al. [5, 8] and Dodson et al. [9].

Many landlocking events have been observed in the threespine stickleback (*Gasterosteus aculeatus*), for which multiple mechanisms have been proposed [17, 49]. Despite the genetic and life-history variation between diadromous and freshwater *G. aculeatus* populations, hybridization does occur [17], though no studies report gene flow between distinct, geographically isolated freshwater populations (although introgressive hybridization between sympatric benthic-limnetic species pairs has occurred [50]). It is possible that with current population dynamics, each landlocked population could eventually lead to distinct taxa, since adaptive radiation has allowed morphological and behavioral variation to accrue in landlocked populations within a short time. Unlike the sympatric sticklebacks, diadromous and landlocked alewife (*Alosa pseudoharengus*) do not typically co-occur, and genetic evidence indicates that many of the landlocked populations are the result of separate isolation events such as damming of a river [18]. Additionally, there are morphological, behavioral and genetic differences between landlocked and diadromous populations that have diverged within the last century [51]. Studies have even started to pinpoint specific functional genes that may play a role in the ability to survive landlocking events [52, 53]. Though no speciation has been recognized in *A. pseudoharengus*, these independent landlocking events (similar to sticklebacks) are the result of multiple transitions out of diadromy [18].

## Conclusion

Complex life histories that require environmental transitions can serve as much more than simple evolutionary stepping stones between different life histories. Diadromy provides species with the ability to enter new environments and diversify, even if the behavior may quickly be lost. While diadromy can act as an evolutionary intermediate between marine and freshwater life histories, it also plays an important role in diversification as well as habitat transitions that are not necessarily between marine and freshwater, but instead marine back to marine or freshwater back to freshwater after diversification from a diadromous life history. With this in mind, continuous studies at the individual, species, and family level are important to understand the mechanism of how and why diadromy is gained and lost. Applying SSE models to other complex life histories may reveal more patterns of biodiversity. Additionally, considering complex life histories that require environmental transitions not only in terms of their costly transitions, but for the advantages of using multiple environments, may allow for new perspectives on multiple topics related to life history evolution.

## Methods

### Species list

The 7822 species of ray-finned fishes (Actinopterygii) used in the recently published phylogeny [30] were each assigned one of three character states: 1) diadromous (*n* = 180), 2) freshwater (*n* = 3998), and 3) marine (*n* = 3600). All species that could not be assigned were classified as “unknown” and dropped from the tree (*n* = 45) (Additional file 1) [6, 54–56]. Diadromous fishes were identified from 28 families (Salmonidae: *n* = 32, Gobiidae: *n* = 19, Anguillidae: *n* = 18, Clupeidae: *n* = 17, Acipenseridae: *n* = 16, Eleotridae: *n* = 14, Osmeridae: *n* = 9, Galaxiidae: *n* = 7, Cottidae: *n* = 6, Achiridae: *n* = 5, Mugilidae: *n* = 5, Cyprinidae: *n* = 4, Retropinnidae: *n* = 4, Salangidae: *n* = 4, Ariidae: *n* = 2, Gasterosteidae: *n* = 2, Moronidae: *n* = 2, Pleuronectidae: *n* = 2, Rhyacichthidae: *n* = 2, Tetraodontidae: *n* = 2, Cheimarrichthyidae: *n* = 1, Gadidae: *n* = 1, Kuhliidae; *n* = 1, Lateolabracidae: *n* = 1, Latidae: *n* = 1, Plecoglossidae: *n* = 1, Pseudaphritidae: *n* = 1, Syngnathidae: *n* = 1). Character states were determined primarily based on information in: Diadromy in Fishes [6], Fishbase [54], Fishes of the World [55], and Encyclopedia of Life [56]. Species considered as euryhaline, or those that can tolerate a large range of salinity including freshwater, were assigned character states based on their dominant life strategy. *Bairdiella chrysoura*, for example, is listed as marine, freshwater, and brackish in Fishbase, but since the species is described as moving “to the nursery and feeding areas in estuaries during summer months and sometimes enters freshwaters,” [54], *B. chrysoura* was listed as a marine species. When inadequate information was available on the aforementioned sources, other primary literature was used in considering salinity levels during breeding, distribution, and salt tolerance, to better assign species to each of the three categories (additional sources used for character assignment: [23, 24, 57–76]). Additionally, the species listed as both marine and freshwater were confirmed via peer-reviewed evidence, such as in the case of *Takifugu poecilonotus,* where studies confirmed diadromy [77]. Species such as *Alosa pseudoharengus* [18], and many of the freshwater eels [78] with both diadromous and non-diadromous individuals/populations, were characterized as diadromous since the behavior is dominant within these species. This was preferable to removing or duplicating these species which would alter measures of speciation and transition between the states.

### Phylogenetic model selection

Species were assigned character states F (freshwater), M (marine), or D (diadromous) for three-state MuSSE analysis. MuSSE gives estimates for speciation (λ), extinction (μ), and transition between states (*q*_FD;_ in this example from state F to state D). Net diversification rates (*r*) were calculated by subtracting extinction rates from speciation rates (*r* = λ-μ). The MuSSE analysis estimates 12 parameters: λ_F_, λ_M_, λ_D_, μ_F_, μ_M_, μ_D_, *q*_FM_, *q*_FD_, *q*_MD_, *q*_MF_, *q*_DF_, and *q*_DM_. Testing different hypotheses was done by putting constraints on specific parameters. For example, to test the hypothesis that all transitions between marine and freshwater have to go through diadromy as shown in Fig. 2I, constraints were put on transition between marine and freshwater in both directions (*q*_FM_ = *q*_MF_ = 0) and on speciation or extinction in diadromy (λ_D_ = μ_D_ = 0). All remaining parameters (λ_F_, λ_M_, μ_F_, μ_M_, *q*_FD_, *q*_MD_, *q*_DF_, and *q*_DM_) were estimated by MuSSE. Looking at Fig. 2III, constraints were put on transitions making estimates of marine to diadromy and diadromy to freshwater equal, as well as freshwater to diadromy and diadromy to marine (*q*_MD_ = *q*_DF_ and *q*_FD_ = *q*_DM_) with all remaining state free to vary. State Stability (*S*) was calculated as one minus the probability of extinction of that state minus the probability of transition out of that state to all other states (*S*_i_ = 1– μ_i_ – *q*_ij_ – *q*_ik_). Similar to other SSE models (e.g., BiSSE, HiSSE, GeoSSE), rates (λ, μ and *q*) are each estimated independently using likelihood starting at the tips of the tree and working back in time along each branch in small time increments. Estimates for each parameter are compiled at nodes until likelihoods have been calculated to the root. For a full description of parameter estimation see Maddison et al. [79], FitzJohn [36] and Ng and Smith [80].

MuSSE analyses were run in R [81] using the “diversitree” package [36]. The correction for incomplete sampling in the tree, the *sampling.f* = (.26, .21, .46) function, was used to inform the program of the percent of freshwater (3998/15,170) and marine (3600/16,764) species based on published estimates [82]. Because recognized non-diadromous species occasionally exhibit diadromous behavior, the true number of species that are diadromous remains unknown. The percent of diadromous species used (104/223) was calculated based on the inventory provided in “Diadromy in Fishes,” [6] where only 104 of the 223 species listed were present in the phylogeny. It is important to note that these percentages are representative of the complete phylogeny across each state starting at the root node. There may be some groups that are fully represented and some that are not represented at all. This may be especially pertinent to radiations such as Sicydiinae gobies or cichlids that have lower than average representation compared to Salmonids that have higher than average representation. A total of 27 models were compared using the MuSSE model (Additional file 2). Akaike information criterion (AIC) scores were calculated from the log likelihood value outputs from each model. ∆AIC was calculated by subtracting the lowest AIC value from each AIC score. Akaike weights (*w*_*i*_) for each model was calculated. Bayesian estimates of the parameters using MCMC, as implemented with the *mcmc* function in DIVERSITREE, were obtained with runs of 10,000 steps. Additionally, to test robustness of character state assignment, models were run with all 45 “unknown” species placed completely in marine or completely in freshwater. Results presented in Table 1 and Additional file 1 do not include these species. All models were run using the single best fit tree reported by Rabosky et al. [35]. No analyses using suboptimal, yet highly supported trees with variation in tree topology or branch lengths were used because no additional topologies were made available for analysis. Additional tree topologies and variation in branch lengths could change the estimated rates produced by all models.

Estimates of extinction rates using phylogenies have been suggested to be inaccurate [83], however, studies have shown that in certain situations phylogenies can be good tools for extinction estimates [41, 84]. As pointed out by Beaulieu and O’Meara [84], using a tree with a large number of taxa (> 100) should be adequate for rate estimations involving extinction, as long as the variation in birth rate is not exceptionally high (also see Rabosky [85]). To see if varying extinction rates affected other parameters, models were compared with all extinction rates constrained to be equal (μ_F_ ~ μ_M_ ~ μ_D_). Parameter estimates from this model resulted in speciation and transition rates similar to those observed in the best fit models (Table 1).

In some cases, an additional characteristic might be nested within the trait of interest resulting in a skewed rate estimation. For example, if temperate diadromous species have a higher speciation rate then tropical diadromous species, or if particular groups or types of diadromy differ from the rest of the diadromous fishes, then they can skew the rate for the diadromous group as a whole when tested in the traditional BiSSE and MuSSE models. Testing for a hidden state can show that other characteristics of the species are important in understanding the evolutionary rates (for a more detailed discussion see Beaulieu and O’Meara [86]). To test for a “hidden state” in diadromy, Hidden State Speciation Extinction models (HiSSE) [86] were also performed. HiSSE assigns a likelihood of each species within a state to a sub state (e.g., “diadromy-observed” and “diadromy-hidden”). HiSSE is a two-state model and was used to test for hidden rates within diadromous and non-diadromous fishes. HiSSE models without hidden states are identical to Binary State Speciation Extinction models (BiSSE) and were also run. While the two state models do not allow for testing the hypotheses of whether diadromy is an intermediate state, identifying a potential hidden state may reveal that a few select taxa are skewing the rates reported in the MuSSE analysis. Using the *MarginRecon* function with the best fit model parameters, all diadromous species were assigned to the diadromy-observed state with > 98% likelihood, with < 2% likelihood of being in a diadromy-hidden state, suggesting no hidden state in diadromy. As a result, models were run with no hidden state for diadromous fishes.

To support the transition rate estimation reported in MuSSE, *fitDiscrete* function in geiger 2.0.3 [87] was also used to estimate transition rates in both a two state (diadromous / nondiadromous) and three state (diadromous / marine / freshwater) data set. Models were compared using AIC. For both two and three-state situations, equal-rate (ER), symmetric (SYM), all rates different (ARD), and meristic (meristic) models were tested. The ER model constrains all transitions rates to be equal (*q*_FM_ = *q*_FD_ = *q*_MD_ = *q*_MF_ = *q*_DF_ = *q*_DM_), SYM constrains rates between two states to be equal (*q*_FM_ = *q*_MF_) but they are free to vary between different states ([*q*_FM_ = *q*_MF_] ≠ [*q*_FD_ = *q*_DF_] ≠ [*q*_DM_ = *q*_MD_]), ARD allows each rate to vary as separate parameter (*q*_FM_ ≠ *q*_FD_ ≠ *q*_MD_ ≠ *q*_MF_ ≠ *q*_DF_ ≠ *q*_DM_), and meristic requires transitions to occur in a stepwise fashion (i.e., M➔D➔F➔D➔M, but not M➔F).

### Model adequacy testing

To test for model adequacy, trees were simulated with the *tree*.*musse* function in the diversitree package [36] incorporating the 12 MuSSE output parameters from the best fit model, with the ancestral state set as marine, 500 trees were simulated to 7778 tips. All trees were simulated with each state being assigned a single estimation of λ and μ that remained consistent throughout the tree. This does not allow the simulation to incorporate mass extinctions or adaptive radiations that have been recorded in the true phylogeny of fishes, and can result in variation of λ and μ at particular locations of the tree. The distribution of the percent of marine, freshwater and diadromous fishes from the 500 simulations were compared to the original percent of taxa in each group. These simulations serve two purposes. First, they validate the MuSSE model by showing the output parameters simulate what is observed in nature. Secondly, they address the potential bias of uneven sampling in the phylogeny used in the study. Showing that results from the tree are representative of what is observed in nature implies that the tree adequately represents the true tree of fishes.

## Additional files

Additional file 1: Table of Species character state assignment. (XLS 832 kb)

Additional file 2: Model constraints for all MuSSE analyses. (DOCX 18 kb)

Additional file 3: Output parameters for optimal model in MuSSE analyses with ambiguous species assigned to marine and freshwater. (DOCX 16 kb)

Additional file 4: Models constraints for HiSSE and BiSSE analysis. (DOCX 24 kb)

## Data Availability

All data generated or analyzed during this study are included in this published article and its supplementary information files.

## Abbreviations

AIC: Akaike information criterion
ARD: All rates different
BiSSE: Binary State Speciation and Extinction
ER: Equal-rate
HiSSE: Hidden State Speciation and Extinction
MuSSE: Multiple State Speciation and Extinction
SYM: Symmetric
