# Supplementary material for: Evolutionary patterns of diadromy in fishes: more than a transitional state between marine and freshwater
Source: BMC Evol Biol. 2019 Aug 14;19:168. doi: 10.1186/s12862-019-1492-2 (PMC6694556; doi:10.1186/s12862-019-1492-2)
Supplement: Supplementary file 2 — Model constraints for all MuSSE analyses. (DOCX 18 kb) [file 12862_2019_1492_MOESM2_ESM.docx]

Additional file 2. Model constraints for all MuSSE analyses.

| MuSSE constraints | ∆ AIC | wi |
| --- | --- | --- |
| Full (No constraints) | 0 | 0.38 |
| *q*_FM_ ~ *q*_FD_ | 0.09 | 0.363 |
| *q*_FM_ ~ 0 | 0.79 | 0.256 |
| µ_F_~µ_M_, µ_D_~µ_M_ | 27.45 | 4.16E-07 |
| *q*_FD_ ~ 0 | 84.41 | 1.78E-19 |
| *q*_FM_ ~ 0,µ_F_~µ_M_, µ_D_~µ_M_ | 133.66 | 3.60E-30 |
| µ_F_~µ_M_, µ_D_~µ_M_, *q*_FM_ ~ *q*_FD_ | 205.05 | 1.13E-45 |
| *q*_MF_ ~ *q*_MD_ | 206.66 | 5.06E-46 |
| *q*_DF_ ~ *q*_DM_ | 207.27 | 3.73E-46 |
| *q*_FM_ ~ *q*_MF_ | 208.56 | 1.96E-46 |
| *q*_MF_ ~ 0 | 238.35 | 6.65E-53 |
| µ_F_~µ_M_, µ_D_~µ_M_,*q*_FM_ ~ *q*_FD_, *q*_MF_ ~*q*_MD_, *q*_DF_~*q*_DM_ | 245.31 | 2.05E-54 |
| *q*_MF_ ~ 0, *q*_FM_ ~ 0 | 265.34 | 9.17E-59 |
| *q*_FM_ ~ *q*_DM_ | 279.5 | 7.72E-62 |
| *q*_MD_ ~ *q*_DM_ | 287.69 | 1.29E-63 |
| *q*_FM_ ~ *q*_FD_, *q*_DM_~*q*_FD_ | 307.48 | 6.48E-68 |
| *q*_DM_ ~ 0 | 309.31 | 2.60E-68 |
| *q*_MD_ ~ 0 | 321.33 | 6.37E-71 |
| *q*_FD_ ~ 0, *q*_DM_ ~ 0 | 324.81 | 1.12E-71 |
| *q*_MF_ ~ *q*_DF_ | 421.66 | 1.04E-92 |
| *q*_FD_ ~ *q*_DF_ | 421.69 | 1.03E-92 |
| µ_F_~µ_M_, µ_D_~µ_M_,λ_F_~λ_M_, λ_D_~λ_M_ | 428.28 | 3.80E-94 |
| *q*_FM_ ~ 0, *q*_FD_~*q*_DM_ | 483.08 | 4.79E-106 |
| *q*_DF_ ~ 0 | 569.69 | 7.47E-125 |
| *q*_MD_ ~ 0, *q*_DM_ ~ 0 | 605.94 | 1.00E-132 |
| *q*_MD_ ~ 0, *q*_DF_ ~ 0 | 612.51 | 3.76E-134 |
| *q*_FD_ ~ 0, *q*_DF_ ~ 0 | 815.65 | 2.910E-178 |

Table S2. Model constraints for MuSSE analyses. Constraints involving speciation rate (λ_i_), extinction rate (μ_i_), and transition rates (*q*_ij_) where _i_ and _j_ refer to the original and new states respectively. Akaike weights (wi) were calculated for each model using equation (1). ~ indicates that a rate was set equal to a second rate or to 0.
