# Supplementary material for: Evolutionary patterns of diadromy in fishes: more than a transitional state between marine and freshwater
Source: BMC Evol Biol. 2019 Aug 14;19:168. doi: 10.1186/s12862-019-1492-2 (PMC6694556; doi:10.1186/s12862-019-1492-2)
Supplement: Supplementary file 3 — Output parameters for optimal model in MuSSE analyses with ambiguous species assigned to marine and freshwater. (DOCX 16 kb) [file 12862_2019_1492_MOESM3_ESM.docx]

Additional file 3. Output parameters for optimal model in MuSSE analyses with “unknown” species in either freshwater or marine.

| Unknown Taxa in Freshwater | | |  |  |  |
| --- | --- | --- | --- | --- | --- |
| λ­_F_ | λ_M_ | λ_D_ | µ_F_ | µ_M_ | µ_D_ |
| 2.15E-01 | 1.40E-01 | 4.51E-01 | 1.54E-01 | 1.29E-01 | 1.39E-06 |
| q_FM_ | q_FD_ | q_MF_ | q_MD_ | q_DF_ | q_DM_ |
| 3.51E-04 | 4.79E-04 | 3.51E-04 | 2.81E-03 | 1.17E-02 | 4.83E-01 |
|  |  |  |  |  |  |
| Unknown Taxa in Marine | | |  |  |  |
| λ_F_ | λ_M_ | λ_D_ | µ_F_ | µ_M_ | µ_D_ |
| 2.18E-01 | 1.37E-01 | 4.51E-01 | 1.56E-01 | 1.26E-01 | 5.01E-07 |
| q_FM_ | q_FD_ | q_MF_ | q_MD_ | q_DF_ | q_DM_ |
| 3.44E-04 | 4.29E-04 | 3.44E-04 | 2.90E-03 | 1.08E-02 | 4.86E-01 |

Table S3. Speciation rate (λi), extinction rate (µi), and transition rates (qij) where i and j refer to the original and new states respectively.
