# Supplementary material for: Evolutionary patterns of diadromy in fishes: more than a transitional state between marine and freshwater
Source: BMC Evol Biol. 2019 Aug 14;19:168. doi: 10.1186/s12862-019-1492-2 (PMC6694556; doi:10.1186/s12862-019-1492-2)
Supplement: Supplementary file 4 — Models constraints for HiSSE and BiSSE analysis. (DOCX 24 kb) [file 12862_2019_1492_MOESM4_ESM.docx]

Additional File 4

Models constraints for HiSSE and BiSSE analysis.

| A) | |  |  |
| --- | --- | --- | --- |
|  | HiSSE constraints | ∆ AIC | wi |
|  | pp27 ta6, ea6, tm8 | 0 | 1 |
|  | *pp30 ta6, ea6, tm9* | 100.45 | 1.54014E-22 |
|  | pp5: ta4, ea5, tm2 | 231.54 | 5.269E-51 |
|  | *pp28: ta7. ea7, tm8* | 2878.2 | 0 |
|  | *pp62: ta4, ea4, tm2* | 1929.48 | 0 |
|  | *pp31: ta7, ea7, tm9* | 2970.82 | 0 |
|  | pp32: ta8, ea8, tm9 | 3033.57 | 0 |
|  | *pp19: ta2, ea2, tm6* | 3103.28 | 0 |
|  | *pp22: ta1, ea1 ,tm10* | *3103.28* | 0 |
|  | pp8: ta3, ea2, tm6 | 3107.01 | 0 |
|  | pp29: ta8, ea8, tm8 | 3111.55 | 0 |
|  | pp20: ta3, ea3, tm6 | 3112.23 | 0 |
|  | pp2: ta3, ea3, tm7 | 3309.32 | 0 |
|  | *pp9: ta2, ea3, tm7* | 3322.46 | 0 |
|  | *pp7: ta3, ea2, tm7* | 3390.88 | 0 |
|  | *pp1: ta2, ea2, tm7* | 3398.48 | 0 |
|  | *pp10: ta2, ea3, tm6* | 3643.07 | 0 |
|  | *pp: ta1, ea1, tm1* | 2.00E+10 | 0 |
|  | *pp4: ta5, ea5, tm2* | 2.00E+10 | 0 |
|  | *pp6: ta4, ea5, tm12* | 2.00E+10 | 0 |

| B) | |  |  |  |  |  |  |  |  |  |  |  |
| --- | --- | --- | --- | --- | --- | --- | --- | --- | --- | --- | --- | --- |
|  |  | net turnover | | | |  |  | extinction fraction | | | |  |
|  |  | ta1=(1,1,1,1) | | | |  |  | ea1=(1,1,1,1) | | | |  |
|  |  | ta2=(1,1,0,0) | | | |  |  | ea2=(1,1,0,0) | | | |  |
|  |  | ta3=(1,2,0,0) | | | |  |  | ea3=(1,2,0,0) | | | |  |
|  |  | ta4=(1,2,3,4) | | | |  |  | ea4=(1,2,3,4) | | | |  |
|  |  | ta5=(1,2,1,2) | | | |  |  | ea5=(1,2,1,2) | | | |  |
|  |  | ta6=(1,2,3,0) | | | |  |  | ea6=(1,2,3,0) | | | |  |
|  |  | ta7=(1,2,1,0) | | | |  |  | ea7=(1,2,1,0) | | | |  |
|  |  | ta8=(1,1,1,0) | | | |  |  | ea8=(1,1,1,0) | | | |  |
|  |  |  |  |  |  |  |  |  |  |  |  |  |
|  | transition rate matrix | | | | | | | | | | | |
|  | tm1 | | (0A) | (1A) | (0B) | (1B) | tm8 | | (0A) | (1A) | (0B) | (1B) |
|  |  | (0A) | NA | 4 | 7 | 10 |  | (0A) | NA | 3 | 5 | 0 |
|  |  | (1A) | 1 | NA | 8 | 11 |  | (1A) | 1 | NA | 6 | 0 |
|  |  | (0B) | 2 | 5 | NA | 12 |  | (0B) | 2 | 4 | NA | 0 |
|  |  | (1B) | 3 | 6 | 9 | NA |  | (1B) | 0 | 0 | 0 | NA |
|  |  |  |  |  |  |  |  |  |  |  |  |  |
|  | tm2 | | (0A) | (1A) | (0B) | (1B) | tm9 | | (0A) | (1A) | (0B) | (1B) |
|  |  | (0A) | NA | 3 | 5 | 0 |  | (0A) | NA | 3 | 4 | 0 |
|  |  | (1A) | 1 | NA | 0 | 7 |  | (1A) | 1 | NA | 0 | 0 |
|  |  | (0B) | 2 | 0 | NA | 8 |  | (0B) | 2 | 0 | NA | 0 |
|  |  | (1B) | 0 | 4 | 6 | NA |  | (1B) | 0 | 0 | 0 | NA |
|  |  |  |  |  |  |  |  |  |  |  |  |  |
|  | tm6 | | 0 | 1 |  |  | tm10 | | (0A) | (1A) | (0B) | (1B) |
|  |  | 0 | NA | 2 |  |  |  | (0A) | NA | 1 | 4 | 0 |
|  |  | 1 | 1 | NA |  |  |  | (1A) | 1 | NA | 0 | 6 |
|  |  |  |  |  |  |  |  | (0B) | 2 | 0 | NA | 7 |
|  | tm7 | | 0 | 1 |  |  |  | (1B) | 0 | 3 | 5 | NA |
|  |  | 0 | NA | 1 |  |  |  |  |  |  |  |  |
|  |  | 1 | 1 | NA |  |  |  |  |  |  |  |  |

1. Models with ∆AIC and model weights for HiSSE and BiSSE models. B) Net turnover (ta), Extinction fractions (ea), and transitions matrices (tm) used in models. For Net turnover and Extinction fractions list represent (0A, 1A, 0B, 1B) where 0 and 1 are non-diadromous and diadromous and A and B represent observed and hidden rates. ta2, ta3, ea2, ea3, tm6 and tm7 are used in models with no hidden states. Ta6, ta7, ta8, ea6, ea7, ea8, tm8, and ta9 are used in models where a hidden state is observed in non-diadromous, but not diadromous fishes.
